# LncRNA NR_027471 Functions as a ceRNA for miRNA-8055 Leading to Suppression of Osteosarcoma by Regulating the Expression of TP53INP1

**DOI:** 10.3389/fonc.2020.563255

**Published:** 2020-09-29

**Authors:** Jiajia Chen, Wujun Miao, Saishuai Yang, Mengchen Yin, Jianning Zhao, Dianwen Song

**Affiliations:** ^1^Department of Spine Surgery, The Second Affiliated Hospital of Nantong University, Nantong, China; ^2^Department of Orthopedics, Shanghai General Hospital of Nanjing Medical University, Shanghai, China; ^3^Department of Orthopedics, School of Medicine, Jinling Hospital, Nanjing University, Nanjing, China; ^4^Department of Anesthesiology, The Second Affiliated Hospital of Nantong University, Nantong, China; ^5^Department of Orthopaedics, LongHua Hospital, Shanghai University of Traditional Chinese Medicine, Shanghai, China; ^6^Department of Orthopedics, Shanghai General Hospital, School of Medicine, Shanghai Jiaotong University, Shanghai, China

**Keywords:** osteosarcoma, TP53INP1, lncRNA, miR-8055, proliferation

## Abstract

Osteosarcoma is a malignancy with high aggressiveness and poor prognosis, which occurs mainly in children. The therapeutic strategy against osteosarcoma includes surgery combined with chemotherapy and radiotherapy. Although the treatment of osteosarcoma has been improved in recent years, there is a large proportion of patients with incurable osteosarcoma. Investigation of the mechanism of osteosarcoma progression would be of great help in discovering therapeutic targets for this disease. Long non-coding RNAs play critical roles in the pathogenesis of different types of cancer. The current study showed that long non-coding RNA NR_027471 was downregulated in osteosarcoma cells. *In vitro* and *in vivo* studies indicated that upregulation of NR_027471 impeded the viability, proliferation, and invasion of osteosarcoma, as well as induced cell cycle arrest at G1. In addition, binding of miR-8055 to NR_027471 was demonstrated, thereby influencing the expression of tumor protein p53 inducible nuclear protein 1 (TP53INP1). Knockdown of NR_027471 promoted epithelial–mesenchymal transition by inhibiting E-cadherin and increasing the expression of zinc finger E-box-binding homeobox 1 (ZEB1), Snail, and fibronectin. These results suggested that overexpression of NR_027471 upregulated TP53INP1 by sponging to miR-8055, leading to suppression of osteosarcoma cell proliferation and progression.

## Introduction

Osteosarcoma is the most common malignancy of bone (1). It is predominantly diagnosed in children and adolescents aged 10–25 years (2). The current treatment of osteosarcoma involves the combination of surgical resection with radiotherapy and chemotherapy. Although the treatment of osteosarcoma has improved in recent years, the survival rate and prognosis of such patients remain poor (3). Identification of the mechanisms underlying the progression of osteosarcoma would help to find promising therapeutic strategies and ameliorate clinical outcomes.

Previous studies have revealed that non-coding DNA accounts for the majority of the human genome, and this is transcribed into non-coding RNA (4). Long non-coding RNAs (lncRNAs) are defined as transcripts longer than 200 nucleotides without evident protein-coding function (5). It has been shown that lncRNAs regulate biological functions, including cell growth, differentiation, progression, and apoptosis (6). Dysregulation of lncRNA expression is associated with different types of cancer, including gastric (7), colon (8), lung (9), and breast (10) cancer.

The aim of the present study was to elucidate the biological functions of the lncRNA NR_027471 in osteosarcoma, as well as the underlying molecular mechanisms. This study may improve our understanding of the role of NR_027471 in osteosarcoma and aid the development of treatment strategies, resulting in decreased recurrence rates and increased survival rates in patients with this disease.

## Materials and Methods

### Cell Culture

Osteosarcoma cell lines (U2OS, Saos-2, MG-63, and HOS) were cultured in Dulbecco's modified Eagle's medium (DMEM) supplemented with 10% fetal bovine serum, 100 U/ml penicillin, and 100 mg/ml streptomycin. The human fetal osteoblast cell line (hFOB1.19) was cultured in medium with DMEM/F-12 (1:1) containing 10% fetal bovine serum and 0.3 mg/l G418. Human foreskin fibroblast-1 (HFF-1) cells and human bone marrow stem cells (hBMSCs) were maintained in DMEM supplemented with 10% of fetal bovine serum, 100 U/ml penicillin, and 100 μg/ml streptomycin (all from Gibco, Carlsbad, CA, USA). All these cell lines were cultured at 37°C in a humidified incubator containing 5% CO_2_.

### Cell Transfection

Transfections were performed using Lipofectamine®3000 (Invitrogen; Thermo Fisher Scientific, Inc.) according to the instructions provided by the manufacturer. For overexpression, the NR_027471 overexpression vector was established using a pLVX-IRES-puro vector backbone produced by Sangon Biotech Co., Ltd. For knockdown, two shRNAs targeting NR_027471 were purchased from Shanghai GeneChem Co., Ltd. (Shanghai, China). The miR-8055 mimics and inhibitor were purchased from Genepharma (Shanghai, China).

### RNA Isolation and Quantitative Reverse Transcription-Polymerase Chain Reaction (PCR)

The total RNA of cells was extracted using the Trizol reagent (ThermoFisher, Shanghai, China #15596018), and the RNA concentration was detected by Nanodrop (ThermoFisher, Shanghai, China). RNA (1 μg) was used along with the RNA reverse transcription kit. Real-time fluorescent quantitative PCR reaction was performed using the SYBR Green RT-qPCR Master Mix kit, and the sample adding reaction was conducted according to the instructions provided by the manufacturer (Takara, Dalian, China #RR420A). The reaction conditions were as follows: program 1: 95°C, 30 s, 1 cycle; program 2: 95°C, 5 s, 50 cycle, 60°C, 34 s; program 3: 95°C, 5 s, 1 cycle, 65°C, 60 s, 97°C, 1 s; and program 4: 42°C, 30 s, 1 cycle. The relative gene expression was calculated by the 2^−ΔΔCT^ method, and the expression of glyceraldehyde-3-phosphate dehydrogenase was used to normalize the expression of mRNA.

### Construction of pLKO.1-Vectors

The pLKO.1-Vector plasmid were purchased from Addgene. The siRNA target to NR_027471 were designed by BLOCK-iT™ RNAi Designer. The sequence of siRNA with with the highest knockdown efficiency and control siRNA was as follows: NR_027471-siRNA, 5′-TGTTGTTGTTGTTGTTATA-3′; Control-siRNA, 5′-TGTTTGTTGTTGTTTGATA-3′; The top strand 5′-CCGGTGTTGTTGTTGTTGTTATACGAATATAACAACAACAACAACATTTTTTGGTACC-3′; and the bottom strand 3′-CACAACAACAACAACAATATGCTTATATTGTTGTTGTTGTTGTAAAAAAAAACCATGGTTAA-5′ was cloned into pLKO.1-Vector plasmid.

### Cell Migration and Invasion Assays

Cells were inoculated into a six-well culture plate (50,000 cells per well). When the cell grew to 100% confluency, 1 ml of blue gun head was used to perform a scratch. Subsequently, the culture medium and suspension cells were removed, and replaced with serum-free culture medium (0 h). Following 24 h of culture, images were captured. The cell migration rate was calculated by comparing the healing degree between 0 and 24 h.

After 1:1 mixing of DMEM medium and Matrigel, 50 μl of the mixture was evenly spread in the Transwell cell and placed in a 37°C incubator for 45 min. The cells were divided into four groups: pLVX-Vector; pLVX-NR_027471; pLKO.1-Vector; and pLKO.1-NR_027471 groups. The cells (20,000 cells per well) were inoculated into the upper chamber of the Transwell. The upper chamber of the Transwell contained serum-free medium, and 700 μl of medium containing 5% serum was added into the lower chamber. The cells in the upper chamber were removed after being cultured in the incubator for 12 h. The adherent cells in the lower chamber of the Transwell were stained with crystal violet and counted after obtaining images.

### Cell Counting Kit-8 (CCK-8) Assay

Cells were inoculated into a 96-well culture plate (4,000 cells per well). Cells were divided into four groups: pLVX-Vector; pLVX-NR_027471; pLKO.1-Vector; and pLKO.1-NR_027471groups. The activity of cells was detected by CCK-8 at 24, 48, 72, or 96 h after inoculation. CCK-8 solution (10 μl) was added into each pore, and the absorbance at 450 nm was detected after incubation at 37°C for 2 h using an enzyme scale.

### Colony Formation Assay

Cells were inoculated into six-well plates (400 cells per well). The cells were divided into four groups: pLVX-Vector; pLVX-NR_027471; pLKO.1-Vector; and pLKO.1-NR_027471groups. Following inoculation, the culture solution was changed once every 3 days. After 14 days of culture, 4% paraformaldehyde was used for fixation for 15 min. Subsequently, 0.1% crystal violet staining solution was added for staining for 5 min. Phosphate-buffered saline (PBS) was used to remove non-specific staining. The number of clones was calculated after capturing images.

### Flow Cytometry Analysis

To detect the apoptotic rate, the transfected cells were stained with annexin V-fluorescein isothiocyanate and propidium iodide using an annexin V-fluorescein isothiocyanate/propidium iodide apoptosis detection kit (Becton, Dickinson and Company, Franklin Lakes, NJ, USA). The cells were analyzed by a Gallios Flow Cytometer (Beckman Coulter, USA) to quantify the percentage of apoptotic cells. For the cell cycle analysis, cells were analyzed using a Cycletest Plus DNA Reagent kit (Becton, Dickinson and Company) according to the instructions provided by the manufacturer. Following 15 min of incubation with the Cycletest Plus DNA Reagent kit, cells were examined using Gallios Flow Cytometer to quantify the proportion of cells in each stage of the cell cycle (S, G1, and G2/M).

### Luciferase Reporter Assay

Cells were seeded into a 96-well-plate for 24 h and subsequently co-transfected with: pmirGLO-NR_027471-wildtype and miR-negative control; pmirGLO-NR_027471-wildtype and miR-8055; pmirGLO-NR_027471-mutant and miR-negative control; or pmirGLO-NR_027471-mutant and miR-8055 (Promega Corporation, Madison, WI, USA), using Lipofectamine® 3000, respectively. Following 48 h at 37°C, Firefly and *Renilla* luciferase activities were measured using the Dual-Luciferase Reporter assay system (Promega Corporation) according to the instructions provided by the manufacturer. Firefly luciferase activity was normalized to *Renilla* luciferase activity.

### RNA Immunoprecipitation (IP)

The cells were harvested, resuspended in nuclear isolation buffer, and maintained on ice with frequent mixing for 20 min. The nuclei were pelleted by centrifugation at 2,500 g for 15 min. Radioimmunoprecipitation assay (RIPA) buffer was used to resuspend the nuclear pellet, which was split equally into two fractions (for mock and IP). Chromatin was mechanically sheared using a Dounce homogenizer with 15–20 strokes. The nuclear membrane and debris were separated after centrifugation. Anti-MS2b (10 μg) was added to the supernatant (10 mg), which was incubated for 2 h at 4°C. Protein A/G beads (40 μl) were added to the mixture, which was incubated for 1 h at 4°C. The beads were subsequently pelleted and washed in RIPA, followed by washing with PBS. Coprecipitated RNAs were isolated by resuspending the beads in Trizol reagent.

### Western Blotting

The cells were divided into four groups: pLVX-Vector; pLVX-NR_027471; pLKO.1-Vector; and pLKO.1-NR_027471 groups. After the culture supernatant was removed, the cells were washed once with PBS, and 100 μl RIPA lysate containing 1 mmol/l phenylmethylsulfonyl fluoride was added into each pore. The cells were detached using a cell scraper on ice, split on ice for 30 min, centrifuged at 12,000 r/min 4°C for 15 min (the centrifugation radius measured 11 cm). Subsequently, the supernatant was collected and the protein concentration was measured through the bicinchoninic acid method. A sodium dodecyl sulfate-polyacrylamide gel electrophoresis gel (10%) was prepared, and 20 μg of protein was loaded into each pore. Following electrophoresis, 300 mA was transferred to 120 min membrane, the protein was transferred to the polyvinylidene difluoride membrane, 5% bovine serum albumin (BSA) was used for blocking for 1 h. The membrane was incubated with 4% BSA overnight, washed thrice with tris-buffered saline with Tween 20, two was incubated at room temperature for 1 h, re-washed thrice with tris-buffered saline with Tween 20, and visualized using a development solution and an enhanced chemiluminescence developer.

### Bioinformatics Prediction

A differential analysis was performed on the gene data of patients with osteosarcoma (GSE85537), downloaded from the Gene Expression Omnibus (GEO) database (https://www.ncbi.nlm.nih.gov/geo/). The TargetScan website (http://www.targetscan.org/vert_72/) predicted the target gene of miR-8055. The MiRcode website (http://www.mircode.org/) predicted the potential miRNAs bound to NR_027471.

### Tumor Xenograft Experiment

All experimental operations were based on the European Union Directive 2010/63/EU for animal experimentation (http://ec.europa.eu/environment/chemicals/lab_animals/legislation_en.htm). Six-week-old female BALB/c nude mice were purchased from Better Biotechnology Co., Ltd. (Nanjing, China). Transfected cells (1 × 10^7^) were resuspended in 100 μl of PBS and subcutaneously inoculated into the flank of nude mice (*n* = 5 per group). Tumor size was recorded once every week, and tumor volume was calculated using the following formula: tumor volume = (length × width^2^)/2. After 3 weeks, all mice were sacrificed, and the tumor bulks were resected and weighed.

### Immunohistochemistry

The tumor tissue was fixed in 4% paraformaldehyde for 4 h, embedded in paraffin, cut into 10 μm paraffin sections, dewaxed, digested with pepsin for 45 min, incubated with 3% hydrogen peroxide for 15 min, and sealed with 5% BSA for 1 h. Subsequently, the tissue was incubated overnight with the primary antibody, followed by incubation with the secondary antibody for 1 h. The tissues were stained with 3,3′-diaminobenzidine for 10 min, and hematoxylin staining was performed.

### Statistical Analysis

The Student's *t*-test was performed using the SPSS software version 19.0 (IBM Corp.). All data are expressed as the mean ± standard deviation of three independent experiments. *P* < 0.05 indicates a statistically significant difference.

## Results

### LncRNA NR_027471 Was Downregulated in Osteosarcoma Cell Lines

**Figure 1:**
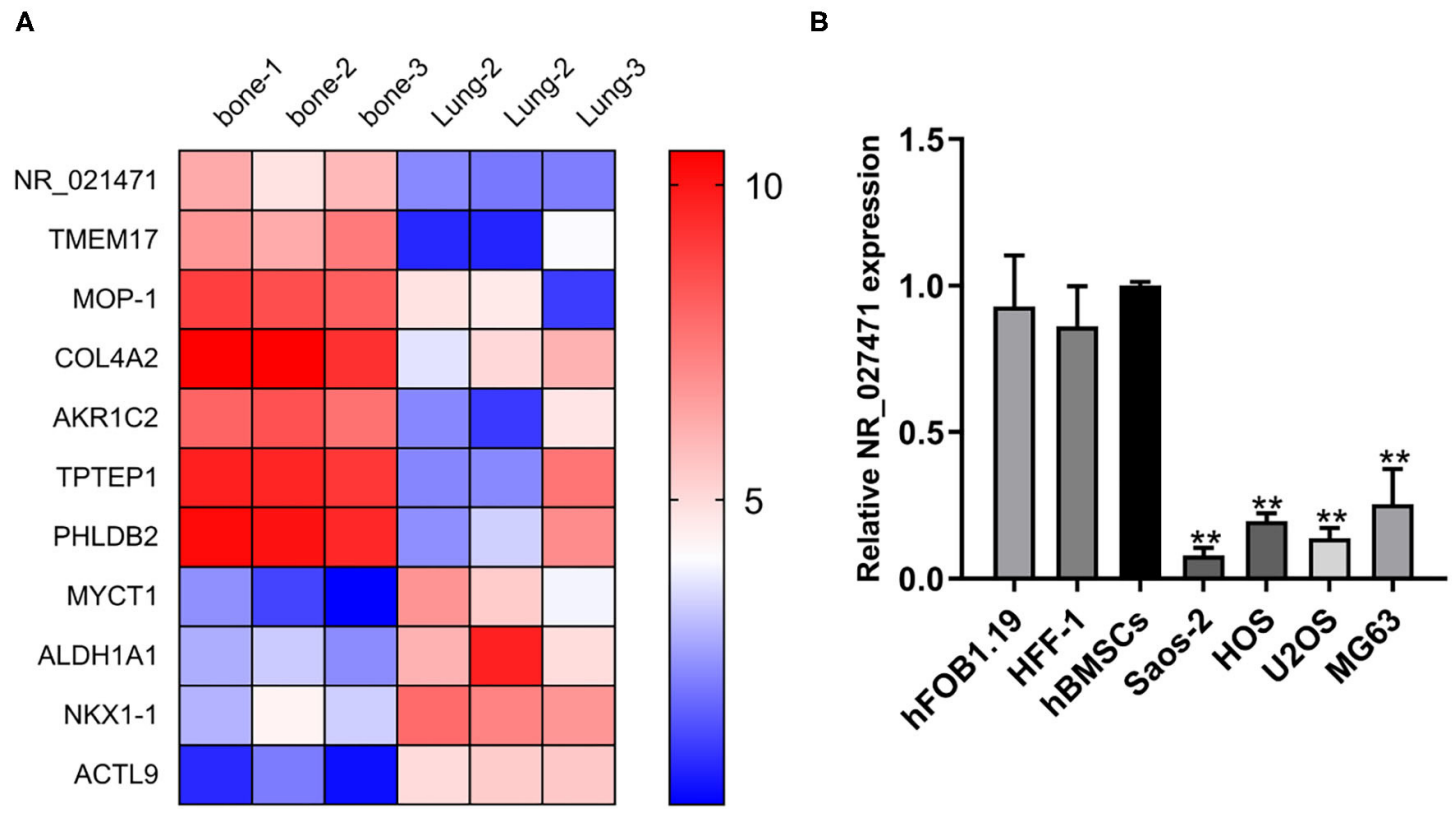
NR_027471 was downregulated in osteosarcoma cells. **(A)** A heatmap showing the expression of different genes at *in situ* lesions or lung metastasis osteosarcoma tissue. **(B)** The expression of lncRNA NR_027471 in hFOB1.19, HFF-1, hBMSCs, and osteosarcoma cell lines (Saos-2, HOS, U2OS, and MG63) was detected by qRT-PCR. Data are expressed as the mean ± SD of three independent experiments.^**^P < 0.01. lncRNA, long non-coding RNA; hFOB, human fetal osteoblast; HFF-1, human foreskin fibroblast-1; hBMSC, human bone marrow stem cell; qRT-PCR, quantitative reverse transcription-polymerase chain reaction; SD, standard deviation.

A bioinformatic analysis from the GEO database showed that NR_027471 was downregulated in lung metastasis compared with *in situ* lesions of osteosarcoma (Figure 1A). The expression of lncRNA NR_027471 was confirmed by quantitative reverse transcription-PCR. The results indicated that NR_027471 was significantly downregulated in osteosarcoma cell lines (U2OS, Saos-2, MG-63, and HOS) compared with hFOB1.19, HFF-1, and hBMSCs (Figure 1B).

### LncRNA NR_027471 Inhibited the Proliferation of Osteosarcoma Cell Lines

**Figure 2:**
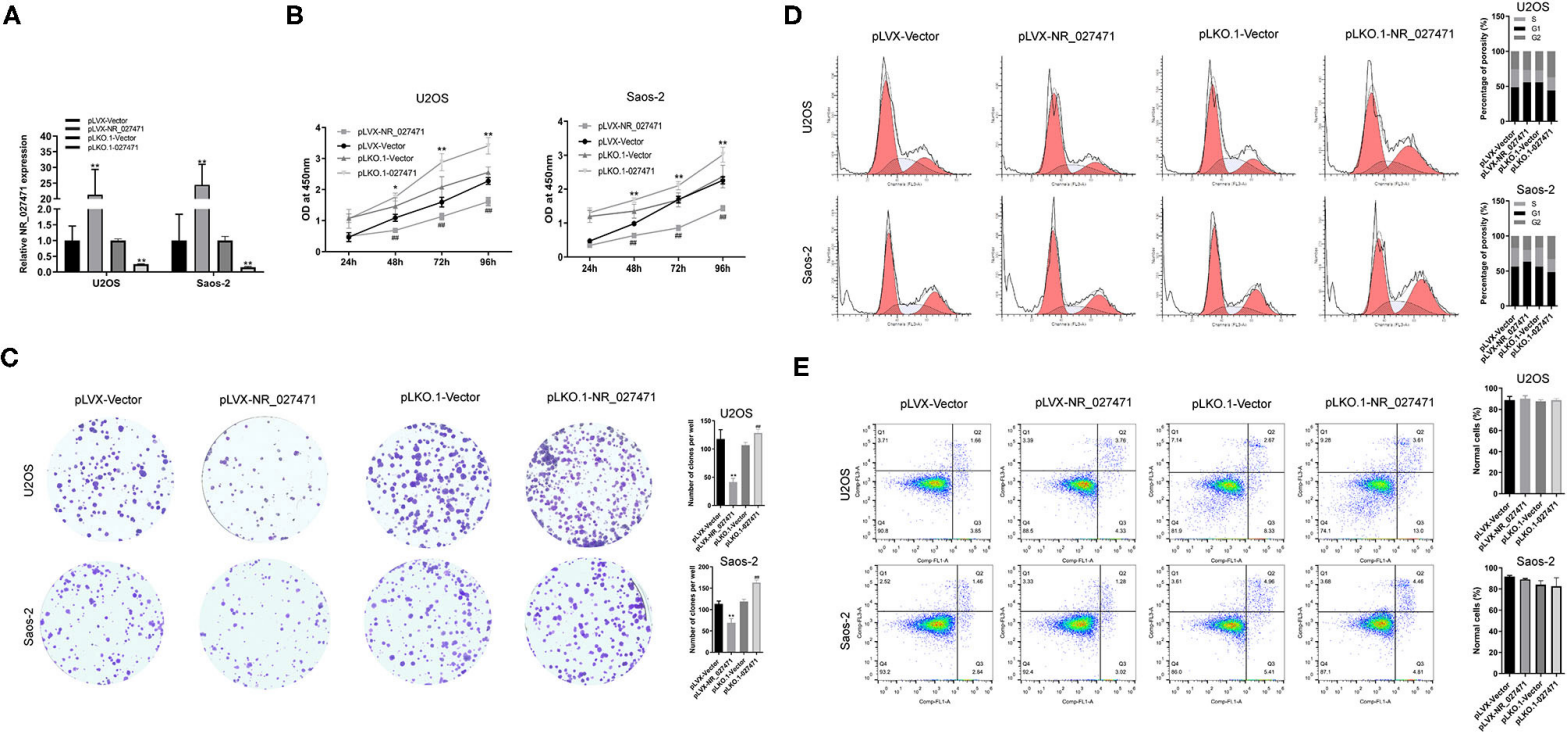
LncRNA NR_027471 inhibited the proliferation of osteosarcoma cells. **(A)** The expression of lncRNA NR_027471 in osteosarcoma cells was determined by qRT-PCR after transfection with pLVX-NR_027471 or pLKO.1-NR_027471. **(B)** The viability of osteosarcoma cells after overexpression or knockdown of lncRNA NR_027471 was determined by CCK-8. **(C)** The proliferation ability of osteosarcoma cells was determined by colony formation assay. **(D)** Cell cycle analysis of osteosarcoma cells after overexpression or knockdown of lncRNA NR_027471 using flow cytometry. **(E)** Apoptotic rate of osteosarcoma cells analyzed by flow cytometry. Data are expressed as the mean ± SD of three independent experiments.^**^P < 0.01 compared with the pLVX-Vector group. ^##^*P* < 0.01 compared with the pLKO.1-Vector group. lncRNA, long non-coding RNA; qRT-PCR, quantitative reverse transcription-polymerase chain reaction; CCK-8, Cell Counting Kit-8; SD, standard deviation.

The expression of lncRNA NR_027471 in U2OS and Saos-2 cells was significantly increased after transfection with pLVX-NR_027471 compared to pLVX-Vector group, whereas that was significantly decreased after transfection with pLKO.1-NR_027471 compared to pLKO.1-Vector group (Figure 2A). CCK-8 analysis showed that osteosarcoma cell viability was suppressed at 48, 72, and 96 h when lncRNA NR_027471 was overexpressed compared to pLVX-Vector group and osteosarcoma cell viability was upregulated at 48, 72, and 96 h with lncRNA NR_027471 knockdown compared to pLKO.1-group (Figure 2B). Colony formation assay was performed to determine the proliferation ability of osteosarcoma cells. The results indicated that osteosarcoma cells transfected with pLVX-NR_027471 had significantly decreased proliferation compared with those transfected with the pLVX-Vector. Osteosarcoma cells transfected with pLKO.1-NR_027471 showed significantly increased proliferation compared with those transfected with the pLKO.1-Vector (Figure 2C). Flow cytometry showed that overexpression of lncRNA NR_027471 induced G1 cell cycle arrest compared to pLVX-NR_027471 group, while knockdown of lncRNA NR_027471 reduced the proportion of cells at G1 compared to pLKO.1-NR_027471 group (Figure 2D). Flow cytometry showed that there was no significant difference between the pLVX-NR_027471 and pLVX-Vector groups regarding the apoptotic rate of osteosarcoma cells. Moreover, there was no significant difference between the pLKO.1-NR_027471 and pLKO.1-Vector groups (Figure 2E).

### LncRNA NR_027471 Inhibited the Migration, Invasion, and Epithelial–Mesenchymal Transition (EMT) of Osteosarcoma Cell Lines

**Figure 3:**
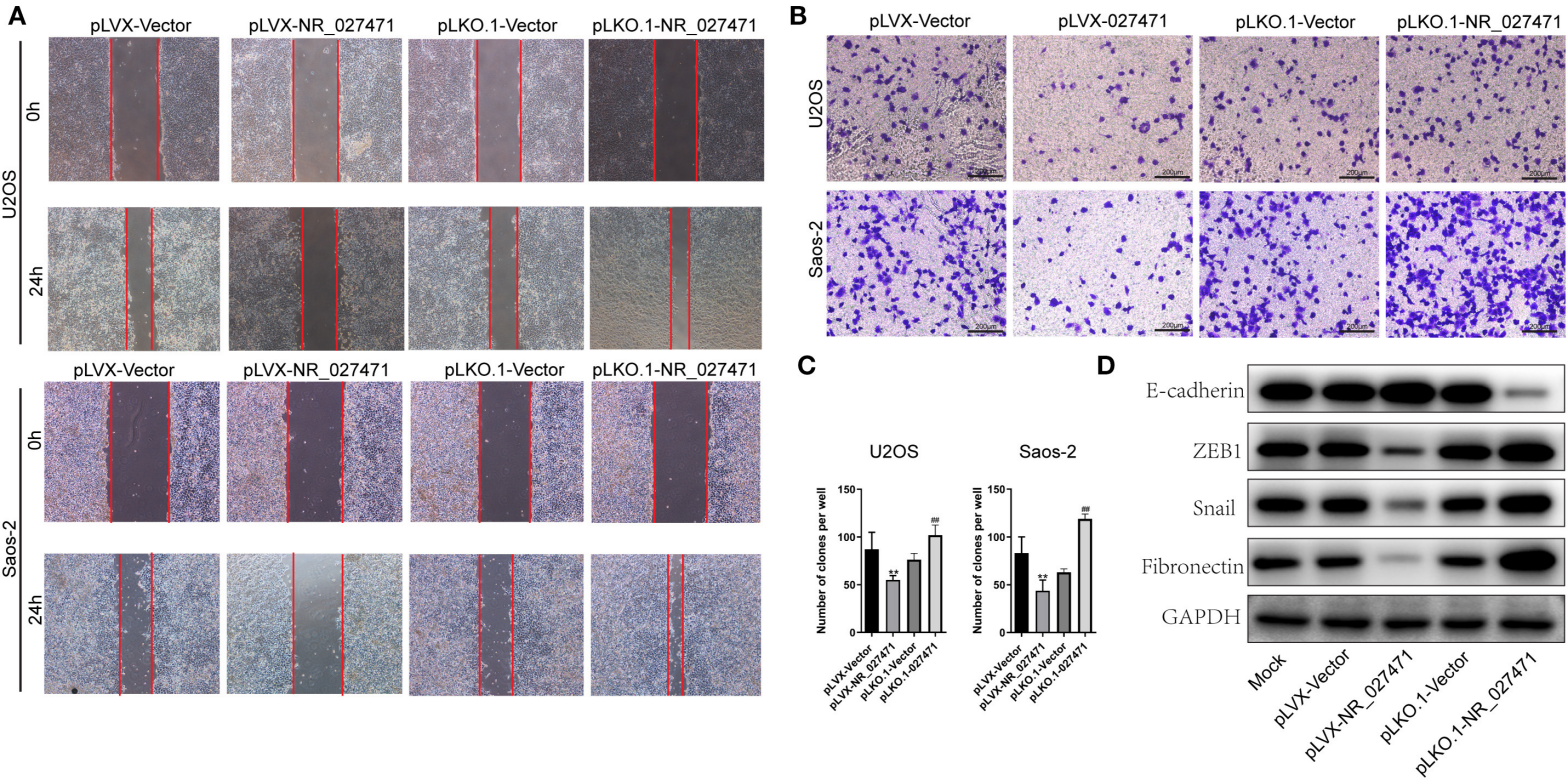
NR_027471 inhibited the migration, invasion, and EMT of osteosarcoma cells. **(A)** The migration ability of osteosarcoma cells after overexpression or knockdown of lncRNA NR_027471 was determined using the scratch assay. **(B)** The invasion ability of osteosarcoma cells after overexpression or knockdown of lncRNA NR_027471 was determined using the Transwell Matrigel assay. **(C)** Quantitative analysis of the invasion ability of osteosarcoma cells after overexpression or knockdown of lncRNA NR_027471 was determined using the Transwell Matrigel assay. **(D)** The protein expression of E-cadherin, ZEB1, Snail, and fibronectin in osteosarcoma cell lines. Data are expressed as the mean ± SD of three independent experiments. ^**^P < 0.01 compared with the pLVX-Vector group. ^##^*P* < 0.01 compared with the pLKO.1-Vector group. EMT, epithelial–mesenchymal transition; lncRNA, long non-coding RNA; ZEB1, zinc finger E-box-binding homeobox 1; SD, standard deviation.

Moreover, the migration ability of osteosarcoma cells was determined by a scratch assay. The experiment revealed that osteosarcoma cells transfected with pLVX-NR_027471 showed decreased migration in comparison with those transfected with the pLVX-Vector. Also, osteosarcoma cells transfected with pLKO.1-NR_027471 showed increased migration vs. those transfected with the pLKO.1-Vector (Figure 3A). The Transwell Matrigel assay showed that the pLVX-NR_027471 group had decreased invasion ability compared with the pLVX-Vector group. Notably, the pLKO.1-NR_027471 group demonstrated increased invasion compared with the pLKO.1-Vector group (Figures 3B,C). The correlation of NR_027471 and EMT was investigated using western blotting. The expression of E-cadherin was upregulated, while that of zinc finger E-box-binding homeobox 1 (ZEB1), Snail, and Fibronectin was downregulated in lncRNA NR_027471-overexpressing osteosarcoma cells compared to pLVX-Vector group (Figure 3D). Reversely, after knockdown of lncRNA NR_027471, the expression of E-cadherin was downregulated, whereas that of ZEB1, Snail, and fibronectin was upregulated in osteosarcoma cells compared to pLKO.1-Vector group (Figure 3D).

### Overexpression of NR_027471 Inhibited the Effect of Osteosarcoma on the Migration and Invasion of Endothelial Cells

**Figure 4:**
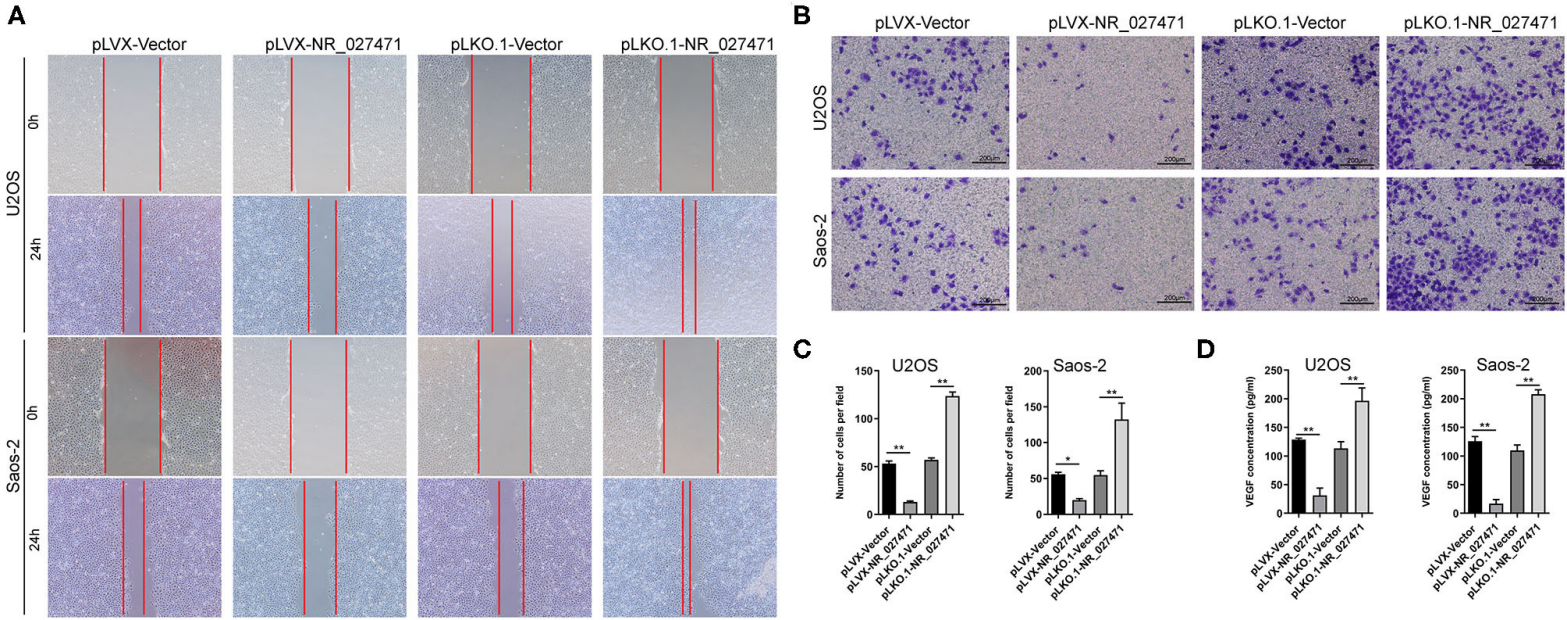
Overexpression of lncRNA NR_027471 inhibited the effect of osteosarcoma on the migration and invasion of endothelial cells. **(A)** Scratch assay and **(B)** Transwell invasion assay performed to analyze the effect of culture supernatant obtained from osteosarcoma cells after overexpression or knockdown of lncRNA NR_027471 on the migration and invasion ability of HUVECs. **(C)** Histogram presenting the number of invasive cells. **(D)** Concentration of VEGF in the supernatant of osteosarcoma cells after overexpression or knockdown of lncRNA NR_027471, analyzed by ELISA. All data are expressed as mean ± SD of three independent experiments. ^*^p < 0.05, ^**^P < 0.01. lncRNA, long non-coding RNA; HUVEC, human umbilical vein endothelial cell; VEGF, vascular endothelial growth factor; ELISA, enzyme-linked immunosorbent assay; SD, standard deviation.

To investigate the impact of NR_027471 on endothelial cells, human umbilical vein endothelial cells (HUVECs) were cultured with culture media derived from osteosarcoma cell with NR_027471 overexpression or knockdown. In the scratch assay, the scratch wound was larger when HUVECs were incubated culture media derived from osteosarcoma cell with NR_027471 overexpression compared that with culture media derived from osteosarcoma cell with pLVX-Vector. In contrast, the scratch wound was smaller when HUVECs were incubated with NR_027471-knockdown media compared with the pLKO.1-Vector group (Figure 4A). The Transwell invasion assay showed that fewer invasive HUVECs were observed at the bottom of the insert following incubation with culture media derived from osteosarcoma cell with NR_027471 overexpression compared that with culture media derived from osteosarcoma cell with pLVX-Vector, whereas more invasive cells were observed after incubation with NR_027471-knockdown media compared with the pLKO.1-Vector group (Figures 4B,C). The concentration of vascular endothelial growth factor in the supernatant of the culture media of U2OS and Saos-2 cells was determined. The results showed that vascular endothelial growth factor was significantly decreased in pLVX-NR_027471 group compared to pLVX-Vector group and increased in pLKO.1-NR_027471 group compared to pLKO.1-Vector group (Figure 4D). Thus, the results indicate that overexpression of NR_027471 inhibits the migration and invasion of endothelial cells.

### LncRNA NR_027471 Regulates the Function of Osteosarcoma by Sponging miR-8055

**Figure 5:**
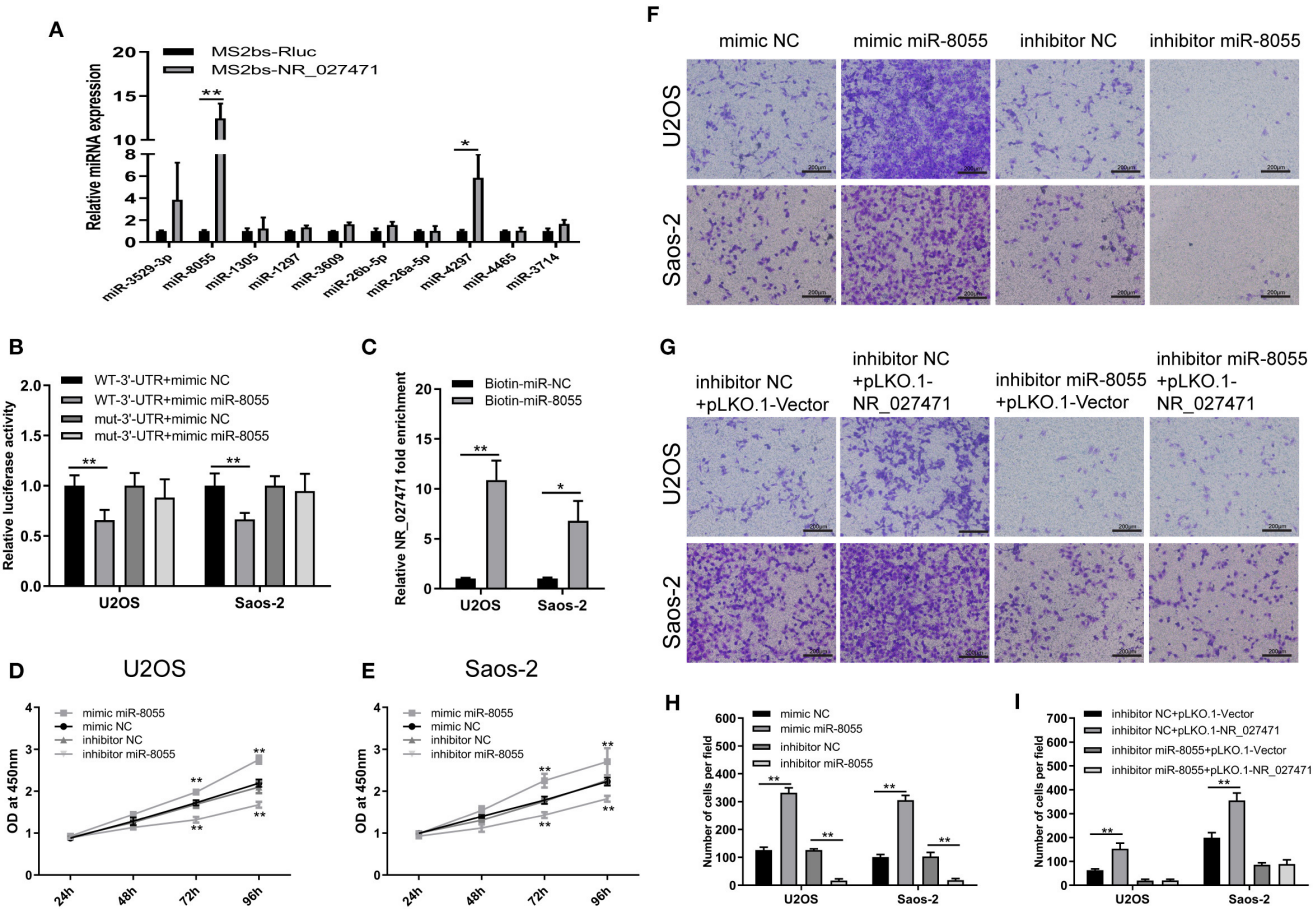
LncRNA NR_027471 regulates the function of osteosarcoma cells by sponging miR-8055. **(A)** MS2b-based RIP assay with anti-GFP antibody (cross-reacting with YFP) in U2OS 48 h after transfection with MS2bp-YFP plasmid along with MS2bs-NR_027471, or MS2bs-Rluc (control vectors). **(B)** Binding of lncRNA NR_027471 and miR-8055 detected using the luciferase assay. **(C)** Binding of lncRNA NR_027471 and miR-8055 using the biotin-miRNA RIP assay. **(D)** Viability of U2OS cells transfected with mimic miR-8055 or inhibitor miR-8055 using CCK-8. **(E)** Viability of Saos-2 cells transfected with a mimic or inhibitor of miR-8055 using CCK-8. **(F)** Transwell invasion assay determined the invasion ability of U2OS and Saos-2 cells transfected with a mimic or inhibitor of miR-8055. **(G)** The Transwell invasion assay determined the invasion ability of U2OS and Saos-2 cells after co-transfection with the miR-8055 inhibitor and pLKO.1-NR_027471. **(H,I)** Histogram presenting the number of invasive cells. Data are expressed as the mean ± SD of three independent experiments. ^**^P < 0.01. lncRNA, long non-coding RNA; RIP, RNA immunoprecipitation; YFP, yellow fluorescent protein; GFP, green fluorescent protein; CCK-8, Cell Counting Kit-8; SD, standard deviation.

The mechanism through which lncRNA NR_027471 regulates osteosarcoma was investigated. The MS2bs-based RNA IP assay indicated that lncRNA NR_027471 combined with miR-8055 (Figure 5A). These results were also confirmed by the luciferase reporter assay (Figure 5B). In addition, we conducted a biotin-miRNA RNA IP assay, which showed that miR-8055 combined with lncRNA NR_027471 (Figure 5C). The CCK-8 assay showed that overexpression of miR-8055 promoted cell viability, whereas its inhibition suppressed cell viability (Figures 5D,E). The Transwell assay revealed that overexpression of miR-8055 promoted cell invasion compared to miRNA mimic NC group, whereas miR-8055 inhibition suppressed cell invasion compared to miRNA inhibitor NC group (Figures 5F,H). Following the inhibition of miR-8055 function by a miR-8055 inhibitor, the impact of NR_027471 on cell invasion was weakened (Figures 5G,I). This finding indicated that the regulatory effect of NR_027471 on invasion was miR-8055-dependent.

### LncRNA NR_027471 Inhibited the Tumor Growth of Osteosarcoma *in vivo* by Modulating the Expression of Tumor Protein p53 Inducible Nuclear Protein 1 (TP53INP1)

**Figure 6:**
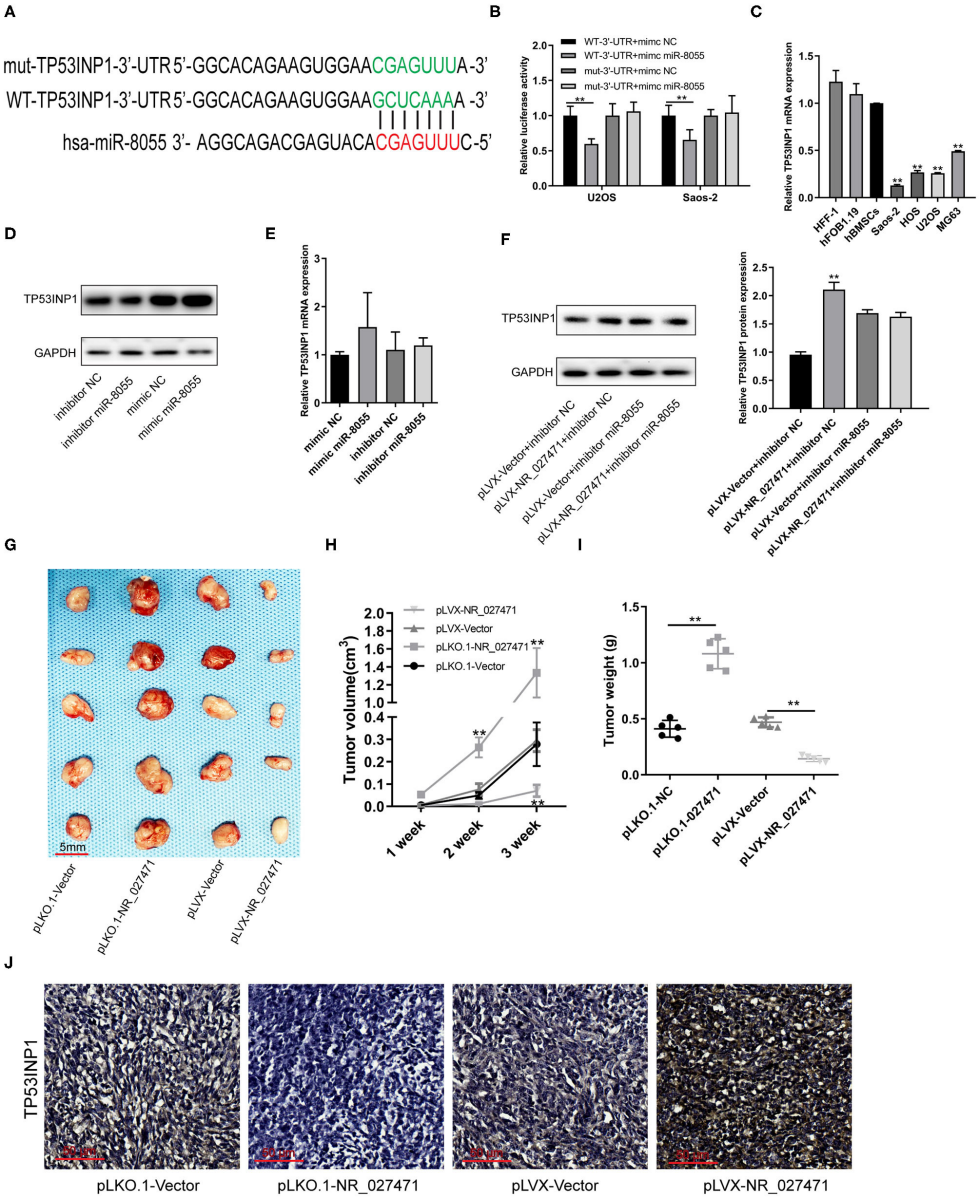
LncRNA NR_027471 inhibited the growth of osteosarcoma *in vivo* by modulating the expression of TP53INP1. **(A)** The WT-3′UTR of TP53INP1 mRNA was predicted as the target of miR-8055 and the mut-3′UTR of TP53INP1 mRNA. **(B)** The mRNA of TP53INP1 was identified as the target of miR-8055 using the luciferase assay. **(C)** qRT-PCR analysis revealed the mRNA expression of TP53INP1 in HFF-1, hFOB1.19, hBMSCs, and osteosarcoma cell lines (Saos-2, HOS, U2OS, and MG63). **(D)** Western blotting analyzed the protein expression of TP53INP1 in U2OS transfected with a mimic or inhibitor of miR-8055. **(E)** qRT-PCR analysis determined the expression of TP53INP1 in U2OS cells transfected with a mimic or inhibitor of miR-8055. **(F)** Western blotting analyzed the protein expression of TP53INP1 in U2OS cell co-transfected with pLVX-NR_027471 and miR-8055 inhibitor. **(G)** The tumor xenografts in nude mice were excised at the end of week 3 (*n* = 5 per group). **(H)** Tumor volume recorded at the end of weeks 1, 2, and 3. **(I)** Tumor weight of xenografts measured at the end of week 3. **(J)** Immunohistochemical detection of the expression of TP53INP1 in tumor tissue. Data are expressed as the mean ± SD of three independent experiments. ^**^P < 0.01. lncRNA, long non-coding RNA; TP53INP1, tumor protein p53 inducible nuclear protein 1; WT, wildtype; 3′UTR, 3′untranslated region; mut, mutant; qRT-PCR, quantitative reverse transcription-polymerase chain reaction; hFOB, human fetal osteoblast; hff, human foreskin fibroblast; hBMSC, human bone marrow stem cell; SD, standard deviation.

A putative binding site between miR-8055 and the 3′ untranslated region (3′UTR) of TP53INP1 mRNA was predicted by TargetScan (Figure 6A). The luciferase reporter assay showed that miR-8055 could combine with the wildtype-3′UTR of TP53INP1 mRNA in osteosarcoma cells, but not with the mutant-3′UTR of TP53INP1 mRNA (Figure 6B). The mRNA expression of TP53INP1 in osteosarcoma cells was confirmed by quantitative reverse transcription-PCR. The expression of TP53INP1 was downregulated in osteosarcoma cell lines (U2OS, Saos-2, MG-63, and HOS) compared with HFF-1, hFOB1.19, and hBMSCs (Figure 6C). The protein expression of TP53INP1 was inhibited following overexpression of miR-8055, whereas it was enhanced after knockdown of miR-8055 (Figure 6D). However, the mRNA expression of TP53INP1was not significantly influenced after overexpression or knockdown of miR-8055 (Figure 6E). Inhibition of the function of miR-8055 using a miR-8055 inhibitor, weakened the impact of lncRNA NR_027471 on the protein expression of TP53INP1 (Figure 6F). This finding indicated that the regulatory effect of lncRNA NR_027471 on TP53INP1 protein was miR-8055-dependent.

Subcutaneous tumor xenografts were developed in nude mice. The tumor volumes were measured at the end of weeks 1, 2, and 3. The analysis showed that the tumor volume was significantly smaller in the pLVX-NR_027471 group vs. the pLVX-Vector group at the end of week 3. In contrast, the tumor volume was significantly larger in the pLKO.1-NR_027471 group vs. the pLKO.1-Vector group at the end of weeks 2 and 3 (Figure 6H). The xenografts were weighed and photographed at the end of week 3 (Figure 6G). The results showed that tumor weight was significantly lower in the pLVX-NR_027471 group vs. the pLVX-Vector group, while it was significantly higher in the pLKO.1-NR_027471 group vs. the pLKO.1-Vector group (Figure 6I). The data indicated that overexpression of NR_027471 inhibits tumor growth of osteosarcoma *in vivo*. Immunohistochemistry showed that the expression of TP53INP1 in the pLVX-NR_027471 group was significantly higher than that observed in the pLVX-Vector group. Moreover, the expression of TP53INP1 in the pLKO.1-NR_027471 group was significantly lower than that measured in the pLKO.1-Vector group (Figure 6J).

## Discussion

In our study, the expression levels of NR_027471 were investigated in osteosarcoma cell lines. Significantly lower expression of NR_027471 was observed in osteosarcoma cell lines compared with osteoblast, fibroblast, and BMSC cell lines. Gain- and loss-of-function experiments demonstrated that NR_027471 overexpression inhibited the cell proliferation and invasion of osteosarcoma cells, and also significantly induced cell cycle arrest at G1 of the osteosarcoma cells. These results suggested a negative association between NR_027471 and progression of osteosarcoma. Moreover, the mechanism through which NR_027471 regulated tumor proliferation and invasion was investigated. NR_027471 positively regulated TP53INP1 by competitively binding with miR-8055. This was the first report investigating the role of NR_027471 in cancer research. The results of this study may improve our understanding of osteosarcoma progression and help to find therapeutic targets against this disease.

LncRNAs play important roles in the formation and progression of osteosarcoma (11–14). LINC00612 functions as a competitive endogenous RNA (ceRNA) for miR-214-5p to promote the proliferation and invasion of osteosarcoma *in vitro* and *in vivo* (15). LncRNA SOX2 overlapping transcript (SOX2-OT) was identified as an oncogene in osteosarcoma cells, regulating the migration, invasion, and expression of cancer stem cell biomarkers. In addition, it was recognized as a prognostic biomarker in patients with osteosarcoma (16). In a study conducted by Zhao et al. (17), BMSC-derived exosomes encapsulated lncRNA PVTl and transported it into osteosarcoma cells, promoting tumor growth and metastasis by inhibiting ubiquitination and upregulating the expression of ERG in these cells. Cong and Jing (18) reported a tumor suppressor, tumor suppressor candidate 7 (TUSC7), which inhibited the proliferation and migration of osteosarcoma cells, promoted cellular apoptosis, and was largely mediated by miR-211. In the present study, NR_027471 was downregulated in osteosarcoma cells and suppressed their proliferation and invasion. NR_027471 plays a tumor suppressive role in osteosarcoma.

LncRNAs exert their function in several manners, including the transcriptional and translational levels (19). At the transcriptional level, lncRNAs may act as ceRNA by binding several miRNAs and inhibiting their activities (20). For instance, lncRNA prostate cancer associated transcript 6 (PCAT6) promote the progression of osteosarcoma through function as ceRNA of miR-185-5p (21). LncRNA HIF1A antisense RNA 2 (HIF1A-AS2) was identified as ceRNA by sponging miR-33b-5p to facilitate cell survival and migration and modulate the expression of sirtuin 6 (SIRT6) in osteosarcoma (22). The bioinformatics analysis performed in the current study revealed that NR_027471 contains a putative binding site for miR-8055, and TP53INP1 was predicted as the target of miR-8055. RNA pull-down and luciferase reporter assays were used to validate the sequence-specific correlation between miR-8055 and NR_027471.NR_027471 in the regulation of TP53INP1 by sponging miR-8055. The present study revealed a novel lncRNA-miRNA target pair that is dysregulated in osteosarcoma cells.

TP53INP1, known as a tumor suppressor, is involved in a series of biological activities. It has been evidenced that miR-182 promotes drug resistance in cisplatin-treated hepatocellular carcinoma cells by downregulating TP53INP1 (23). By antagonizing TP53INP1 and Yes1 associated transcriptional regulator (YAP1), upregulated miR-200a enhances drug resistance in breast cancer (24). In colorectal cancer, miR-221 promoted cell proliferation via the inhibition of autophagy and targeted TP53INP1 (25). In this study, TP53INP1 was predicted and confirmed to be a target of miR-8055. The upregulation of NR_027471 increased the protein expression of TP53INP1. Therefore, both NR_027471 and TP53INP1 appear to play tumor suppressive roles in osteosarcoma.

## Conclusion

In this study, a newly identified regulatory mechanism of the NR_027471/miR-8055/ TP53INP1 axis was systematically studied in osteosarcoma. NR_027471 suppresses the proliferation and invasion of osteosarcoma cells and induces cell cycle arrest at G1. NR_027471 inhibited EMT by increasing E-cadherin and decreasing ZEB1, Snail, and fibronectin. NR_027471 regulates the protein expression of TP53INP1 by sponging miR-8055. This may improve our understanding of epigenetic regulation via NR_027471 and miR-8055 in osteosarcoma and may provide a novel insight into potential therapeutic strategies.

## Data Availability Statement

The datasets generated for this study are available on request to the corresponding author.

## Ethics Statement

The animal study was reviewed and approved by Animal Care and Use Committee of Shanghai General Hospital of Nanjing Medical University.

## Author Contributions

DS and JZ: conception and design: JC, WM, and SY: experiments and data analysis: JZ: intellectual input and supervision: JC, MY, and DS: manuscript writing. All authors approved the final version of the manuscript.

## Conflict of Interest

The authors declare that the research was conducted in the absence of any commercial or financial relationships that could be construed as a potential conflict of interest.
